# A Novel Mechanism of Carvedilol Efficacy for Rosacea Treatment: Toll-Like Receptor 2 Inhibition in Macrophages

**DOI:** 10.3389/fimmu.2021.609615

**Published:** 2021-07-12

**Authors:** Jiawen Zhang, Peiyu Jiang, Lei Sheng, Yunyi Liu, Yixuan Liu, Min Li, Meng Tao, Liang Hu, Xiaoyan Wang, Yanjing Yang, Yang Xu, Wentao Liu

**Affiliations:** ^1^ Department of Dermatology, The First Affiliated Hospital of Nanjing Medical University, Nanjing, China; ^2^ Jiangsu Key Laboratory of Neurodegeneration, Department of Pharmacology, Nanjing Medical University, Nanjing, China; ^3^ Department of Dermatology, The Second Affiliated Hospital of Nanchang University, Nanchang, China; ^4^ Jiangsu Key Laboratory of Oral Disease, Nanjing Medical University, Nanjing, China

**Keywords:** rosacea, carvedilol, macrophage, TLR2, KLK5, cathelicidin

## Abstract

**Background:**

Rosacea, a chronic inflammatory skin disorder etiologically associated with immune cells and the antibacterial peptide cathelicidin LL-37, can be effectively treated by oral carvedilol administration.

**Objective:**

To investigate the molecular mechanisms underlying carvedilol efficacy in rosacea treatment.

**Methods:**

Skin samples of patients with rosacea were subjected to histopathological (hematoxylin and eosin) and immunohistochemical (CD68, Toll-like receptor 2 (TLR2), kallikrein 5, cathelicidin, TNF-α, and IL-1β) evaluation. An *in vivo* murine rosacea-like inflammation model was established by LL-37 intradermal injection with or without carvedilol gavage-based pretreatment. Erythema proportion (Image J) and skin redness (L*a*b colorimetry) were quantified. Murine skin samples underwent pathological examination for inflammatory status and immunofluorescence staining. Murine skin and lipopolysaccharide-stimulated RAW 264.7 cells with or without carvedilol pretreatment were evaluated by quantitative reverse transcription-polymerase chain reaction and western blotting. Clinical facial images of patients were obtained using the VISIA skin analysis system before, 4, and 6 months following oral carvedilol administration.

**Results:**

Rosacea skin lesions exhibited more pronounced inflammatory cell infiltration than peripheral areas, with profound macrophage infiltration and inflammatory cytokines (TLR2, kallikrein 5, cathelicidin, TNF-α, and IL-1β). *In vivo*, carvedilol alleviated inflammation in LL-37 mice, down-regulating TLR2, KLK5, and cathelicidin expression. *In vitro*, carvedilol decreased TLR2 expression in RAW 264.7 cells, further reducing KLK5 secretion and LL-37 expression and ultimately inhibiting rosacea-like inflammatory reactions. Clinical manifestations and facial redness obviously improved during 6-month follow-up with systemic carvedilol administration.

**Conclusion:**

Carvedilol is effective against rosacea, with inhibition of macrophage TLR2 expression as a novel anti-inflammatory mechanism.

## Introduction

Rosacea is a common chronic inflammatory skin disease of the central facial skin, with typical manifestations such as flushing, erythema, papules, pustules, and phymatous changes (1). The prevalence of rosacea was reported to be 5.41% in women and 3.90% in men (2) and varies from 1% to 22% worldwide (3). In 2002, the National Rosacea Society (NRS) assembled an expert committee to develop the first standard classification of rosacea, which was classified into four subtypes and one variant including erythematotelangiectatic, papulopustular, phymatous, ocular, and granuloma, respectively (3). Because rosacea can encompass a multitude of possible combinations of signs and symptoms, in 2018, the NRS suggested that no definite demarcation existed between various subtypes, instead proposing an updated standard classification of rosacea based on phenotypes (4). In particular, fixed centro-facial erythema is considered a diagnostic phenotype of rosacea (1) and erythema is the most commonly observed manifestation among all types of rosacea.

The pathophysiology of rosacea has not yet been completely elucidated. The primary erythematotelangiectatic phenotype is considered to be related to neurovascular inflammatory response (neurovascular inflammation), whereas the papulopustular phenotype is related to both neurovascular inflammation and abnormal innate and adaptive immune responses (3). Cathelicidin LL-37 plays a core role in the innate immunity of rosacea inflammation. Various kinds of external stimulations including ultraviolet rays and microorganisms directly or indirectly increase the activity of kallikrein-related peptidase 5 (KLK5) in the stratum corneum *via* Toll-like receptor 2 (TLR2), cleaving the inactive precursor protein cathelicidin (hcap18) to form the active LL-37 fragment (5, 6). Accordingly, the mechanism of action of some rosacea medications including ivermectin, doxycycline, and azelaic acid is related to the inhibition of kallikrein 5 and cathelicidin expression (7–9).

Various cells of the innate immune system including macrophages, mast cells, and neutrophils contribute to rosacea pathogenesis, with profound macrophage infiltration being observed in all subtypes of rosacea. Buhl et al. (10) performed immunohistochemical staining for CD68 on lesion tissues collected from patients with different rosacea subtypes and found strongly significant increases in CD68^+^ cells (i.e., macrophages) in all subtypes. Macrophages were diffusely distributed in granulomatous areas and interfollicular areas in cases of papulopustular rosacea, whereas a perivascular pattern was mainly observed in the erythematotelangiectatic subtype (10).

Carvedilol, an α1-, β1-, and β2- antagonist, is recommended worldwide by some guidelines and consensuses for the treatment of flushing and persistent erythema in rosacea (11). The mechanism is thought to involve the constriction of smooth muscles in small arterioles by blocking the beta2-adrenergic receptor. Carvedilol can also retard heart function by blocking cardiac beta2-adrenergic receptors, further improving refractory facial flushing and persistent erythema (12).

Inflammation mediated by TLR2, which is widely expressed on macrophages, constitutes an effective therapeutic target in numerous inflammatory diseases. For example, the TLR2/NF-κB pathway in macrophages constitutes a therapeutic target for corneal inflammation (13) and TLR2 is necessary for rhinovirus-induced inflammation, with TLR2 macrophages being sufficient to cause airway inflammation in TLR2-knockout mice (14). The TLR2/KLK5 pathway is also important for rosacea, as serine protease KLK5 activity and cathelicidin expression are highly up-regulated in this disease, promoting skin inflammation (5). Moreover, TLR2 expression is increased and stimulates KLK5 production by keratinocytes in patients with rosacea (6). However, whether the TLR2/KLK5/cathelicidin pathway in macrophages is involved in the mechanism underlying the efficacy of carvedilol in rosacea remains unknown.

Accordingly, in this study, we aimed to investigate the potential effects of carvedilol on TLR2 and inflammatory reactions related to macrophages in rosacea, both *in vivo* and *in vitro*. These findings provide new insights regarding the mechanisms underlying the therapeutic efficacy of carvedilol in rosacea.

## Materials and Methods

### Ethics Statement

All experiments were strictly performed in accordance with the regulations of the ethics committee of the International Association for the Study of Pain and the Guide for the Care and Use of Laboratory Animals (The Ministry of Science and Technology of China, 2006). All animal experiments were approved by the Nanjing Medical University Animal Care and Use Committee and designed to minimize suffering and the number of animals used. All human tissue specimens and clinical pictures of patients with rosacea were obtained from the Department of Dermatology of The First Affiliated Hospital of Nanjing Medical University, and the study was approved by the ethics committee of the First Affiliated Hospital of Nanjing Medical University.

### Clinical Observation of Patients With Rosacea Treated With Oral Carvedilol Only

The clinical characteristics of the four patients with rosacea were recorded. Facial images were obtained using the VISIA^®^ 6.0 Complexion Analysis System (Canfield Scientific Inc., Parsippany, NJ, USA); the red area images were then analyzed using Image J v1.51 and the percentage of erythema was further calculated. Finally, the severity of rosacea was assessed by two blinded senior dermatologists according to the Investigator Global Assessment (IGA) score using a 5-point scale (15).

### Rosacea-Like Inflammation Mouse Model and Grouping

The rosacea-like inflammation mouse model was induced as previously reported (5). Healthy female BALB/c mice (6–8 weeks old) were purchased from Cavens Laboratory Animal Co. Ltd. (Jiangsu, China). Mice were shaved on the back 24 h before injections. The next day, pentobarbital sodium (50 mg/kg) was injected intraperitoneally for anesthesia after weighing. Until completely relaxed, the mice were placed in a prone position on a self-made cystosepiment. LL-37 (50 μL of 320 μM dissolved in phosphate-buffered saline (PBS) was injected intradermally (i.d.) to form a small lump with a diameter of 1 cm.

LL-37 (LLGDFFRKSKEKIGKEFKRIVQRIKDFLRNLVPRTE) was custom synthesized by Sangon Biological Technology (Shanghai, China), at > 99% purity as confirmed by high-performance liquid chromatography. Following injection, the mice were returned to the animal room for normal feeding. The injections were administered four times consecutively each 12 h; the normal control group received 50 μL PBS as an alternative. At 12 h after the last injection, skin inflammation was estimated by observing erythema, erythema percentage, and color difference. At 72 h after the last injection, skin samples were taken for hematoxylin–eosin (HE) staining, immunofluorescence staining, protein extraction, and RNA extraction.

Carvedilol (Sigma, St. Louis, MO, USA) was dissolved in odium carboxymethyl cellulose (CMC-Na) at a concentration of 4 mg/mL and intragastrically (i.g.) administered at a dose of 40 mg/kg once daily for 14 days before LL-37 i.d. injection. The last LL-37 injection was performed on the last day of carvedilol administration (**Figure 2A**). CMC-Na was i.g. administered at a dosage of 0.1 mL/g rather than carvedilol as a negative control. **Figure 2A** described the process of injection and oral administration.

Female BALB/C mice were randomly divided into four groups, (6 mice per group): PBS+CMC-Na (PBS, 50 mL, i.d.+CMC-Na, 0.1 mL/g, i.g.), LL-37+CMC-Na (LL-37, 320 μM, 50 μL, i.d.+CMC-Na, 0.1 mL/g, i.g.), LL-37+carvedilol (LL-37, 320 μM, 50 μL, i.d.+carvedilol, 40 mg/kg, i.g.), and PBS+carvedilol group (PBS, 50 μL, i.d.+carvedilol, 40 mg/kg, i.g.). The mice were photographed 12 h after the last i.d. injection. The degree of erythema was visually evaluated and quantitatively measured as A* value using a LAB colorimeter (CR-10 Plus, Konica Minolta, Tokyo, Japan) and the proportion of erythema in the local area was analyzed using Image J. The CIELAB system is a three-dimensional color-space consisting of three axes. The L* axis is a gray scale with values from 0 (black) to 100 (white). A* is the red/green axis; positive and negative A* represent red and green values, respectively, which correlate with erythema. B* is the yellow/blue axis; positive and negative B* describe the yellow and blue values, respectively, and correlate with pigmentation and tanning.

### Histopathological Analysis of Changes in Skin Samples of Patients With Rosacea and the Rosacea-Like Inflammation Mouse Model

Human tissues were obtained from the facial skin of patients with rosacea. Murine skin was obtained 72 h after the last injection of LL-37. All samples were fixed with formaldehyde, dehydrated with a graded alcohol series, embedded in paraffin, sliced into 5-μm sections, and subjected to HE staining. The pathological manifestations were evaluated and photographed under an optical microscope at 40, 100, and 200× magnification by a blinded observer.

### Cell Culture and Grouping

The RAW 264.7 (2 × 10^5^) mouse monocyte line (ATCC TIB-71; American Type Culture Collection, Manassas, VA, USA) was used as the macrophage line and cultured in Dulbecco’s modified Eagle’s medium (Gibco, Gaithersburg, MD, USA) compounded with 10% fetal bovine serum (Gibco) under humidified 5% CO_2_ at 37°C. The cells were equalized into six-well plates and then treated with lipopolysaccharide (LPS, 1 μg/mL) for 12 h with or without carvedilol (10 μM) pretreatment for 30 min; protein was extracted when the cell density reached 60–70%. This procedure was repeated for RNA collection except using a 6-h LPS treatment; RNA was stored at −80°C for analysis. The cells were also equalized into six-well plates and then treated with lipopolysaccharide (LPS, 1 μg/mL) for 12 h with carvedilol (10 μM) pretreatment for 30 min or TLR2 inhibitor (C29, 50 nM) pretreatment for 1 hour; protein was extracted when the cell density reached 60–70%.

### Measurement of Cell Proliferation

RAW 264.7 cells were seeded in 96-well plates and treated with different concentrations (1, 5, 10, and 20 µM) of carvedilol for 12.5 h. CCK8 solution (APExBIO, Boston, MA, USA) was added at 10 µL per 100 µL medium. After a 2-h incubation under 5% CO_2_ at 37°C, cell proliferation was assessed by measuring the optical density (OD) at 450 nm using a microplate reader (Thermo Fisher Scientific, Waltham, MA, USA).

### Immunohistochemical Analysis

The paraffin sections of human skin were washed with PBS and then incubated with 3% H_2_O_2_ for 10 min. The antibodies including against TLR2 (1 mg/mL diluted by 1:100), KLK5 (1.14 mg/mL diluted by 1:100), cathelicidin (3.44 mg/mL diluted by 1:100), CD68 (0.623 mg/mL diluted by 1:100), TNF-α (0.2 mg/mL diluted by 1:100), and IL-1β (2 mg/mL diluted by 1:100; all from Abcam, Cambridge, UK) were incubated at room temperature for 2 h. After incubation with polymer enhancer for 20 min, the tissue was incubated with polymer enhancer and enzyme-labeled mouse or rabbit polymers. Slides were washed with PBS and fresh diaminobenzidine, counterstained with hematoxylin, antigen retrieval performed using 0.1% HCl, dehydrated with ethanol, cleaned with xylene, and fixed with neutral balata. The results were observed and photographed using a fluorescence microscope and visualized under a light microscope at 200× magnification by a blinded observer. Controls without primary antibodies showed no immunolabeling. Light to dark brown staining indicated a positive result. The stained areas were analyzed using Image J. The average positive percentage of any 10 stained areas was calculated as the staining score.

### Immunofluorescence Staining in Mouse Skin and RAW 264.7 Cells

The paraffin sections of human or mouse skin were flushed with PBS, then sealed with 5% donkey serum containing 0.3% Triton-100 at room temperature for 2 h. After blocking, anti-CD68 (1 mg/mL diluted by 1:100), anti-TLR2 (0.6 mg/mL diluted by 1:100), anti-KLK5 (1.295 mg/mL diluted by 1:100) and anti-IL-6 (0.555mg/ml diluted by 1:100) antibodies (all from Abcam) were incubated overnight at 4°C in a dark wet box. The sections were then incubated with Alexa Fluor 488 or 555 antibodies as the secondary antibody at room temperature for 2 h. Finally, 4′, 6-diamino-2-phenylindole (DAPI, Sigma) was counterstained for 15 min.

RAW 264.7 cells were fixed with 4% paraformaldehyde for 30 min, permeabilized with PBS containing 0.3% Triton-100 for 20 min, and then sealed with 10% donkey serum for 30 min with PBS washes between each step. Next, the cells were incubated with anti-TLR2 (0.6 mg/mL diluted by 1:100), anti-KLK5 (1.295 mg/mL diluted by 1:100), and Alexa Fluor 488 (1:300) or 555 (1:300; Thermo Fisher Scientific) antibodies as primary and red- and green-fluorescently labeled secondary antibodies, respectively. Finally, DAPI was counterstained for 15 min. Images were captured using a Zeiss Axio Scope A1 (Zeiss, Oberkochen, Germany).

### Western Blotting

Samples were collected and dissociated with lysis buffer, then replenished with protease inhibitors and phosphatase inhibitors. Subsequently, the proteins were separated by sodium dodecyl sulfate-polyacrylamide gel electrophoresis and transferred to polyvinylidene fluoride membranes. The membranes were blocked with 5% evaporated milk for 2 h at room temperature, then incubated with antibodies against TLR2 (Abcam), KLK5 (Abcam), cathelicidin (Abcam), and β-actin (Santa Cruz Biotechnology, Dallas, TX, USA) with rocking overnight at 4°C. The membranes were then incubated with the secondary antibodies (Cell Signaling Technology, Danvers, MA, USA) for 2 h at room temperature. Quantity One software (Bio-Rad Laboratories, Hercules, CA, USA) was used for densitometry analysis.

### Pro-Inflammatory Cytokines Production

The levels of pro-inflammatory cytokines, including TNF-α, IL-6 and IL-8, in the cell culture supernatant were measured using the ELISA commercial reagent kits (Yi Fei Xue Biotechnology, Nanjing, China) following the manufacturer’s instructions.

### Reverse Transcription-Quantitative Polymerase Chain Reaction Analysis

After processing the samples, RNA was extracted using TRIzol reagent (Ambion, Austin, TX, USA). According to the determined concentration, 500 ng RNA was transcribed to 10 mL cDNA using a Hiscript II Q RT Supermix Kit (Vazyme, Nanjing, China). Specifically, 5 μL 2 × SYBR qPCR Master Mix (Vazyme), 2 µL primes, 0.5 µL cDNA, and 2.5 µL RNAnase-free ddH_2_O was used for RT-PCR in the Real Time PCR System^®^. The reaction conditions were as follows: 1 cycle of 95°C for 30 s, 40 cycles of 95°C for 10 s, and 1 cycle of 60°C for 30 s. All samples were replicated in duplicate and the relative expression was calculated using the formula 2−ΔΔCt, ΔCt = Ct gene − Ct GAPDH. Primer sequences (Invitrogen-Thermo Fisher Scientific, Shanghai, China) were as follows (5’-3’): *TLR2*, TCTAAAGTCGATCCGCGACAT (forward) and CTACGGGCAGTGGTGAAAACT (reverse); *KLK5*, ATGGGCAATGGCTACCCTG (forward) and GTTCGGTTCCAGAGGGGTT (reverse); *CAMP*, GCTGTGGCGGTCACTATCAC (forward) and TGTCTAGGGACTGCTGGTTGA (reverse); *ITGAM*, CCATGACCTTCCAAGAGAATGC (forward) and ACCGGCTTGTGCTGTAGTC (reverse); *ITGB2*,CAGGAATGCACCAAGTACAAAGT (forward) and CCTGGTCCAGTGAAGTTCAGC (reverse); *TNFA*, CTGAACTTCGGGGTGATCGG (forward) and GGCTTGTCACTCGAATTTTGAGA (reverse); *IL6*, TAGTCCTTCCTACCCCAATTTCC (forward) and TTGGTCCTTAGCCACTCCTTC (reverse); *IL8*, CGGGGTTTTCGCTGTACCT (forward) and GGAATCACATTGGCGTTCCTC (reverse); *IL1B*, GCAACTGTTCCTGAACTCAACT (forward) and ATCTTTTGGGGTCCGTCAACT (reverse); and *GAPDH*, AGGTCGGTGTGAACGGATTTG (forward) and TGTAGACCATGTAGTTGAGGTCA (reverse).

### Statistical Analysis

All *in vitro* experiments were performed at least three times in triplicate and data were statistically analyzed using GraphPad Prism version 6.0 (LaJolla, CA, USA). The values in the figures are expressed as the means ± SEM. A paired t-test was used for statistical analysis of the results, with P < 0.05 considered statistically significant.

## Results

### Obvious Inflammatory Reaction And Profound Infiltration of Macrophages in Skin Samples of Patients With Rosacea

**Figure 1:**
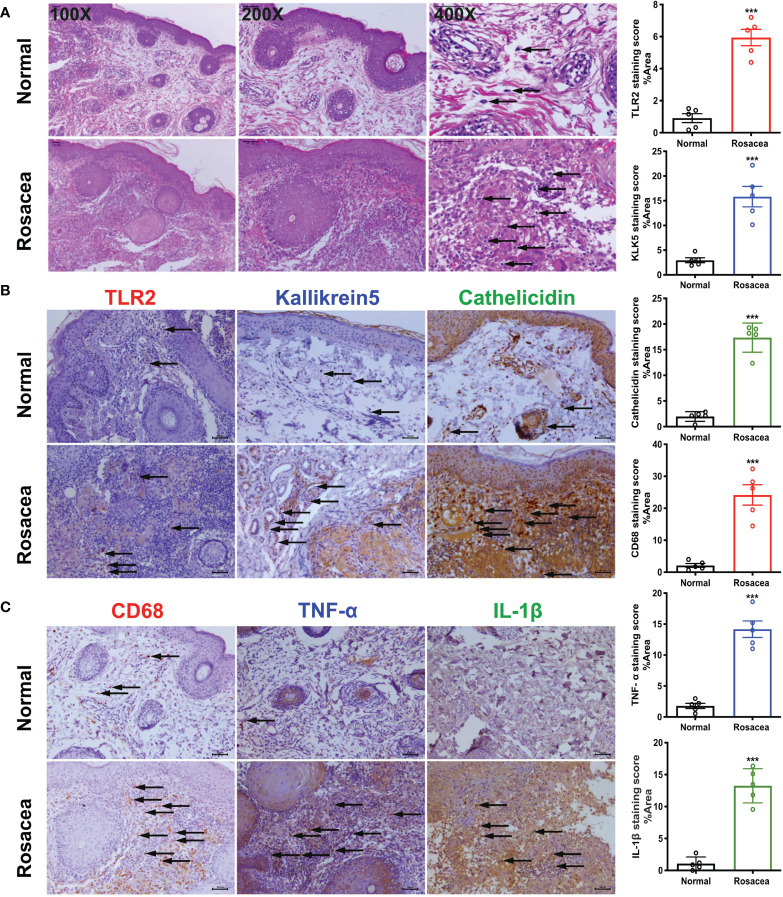
Inflammatory cells especially macrophages are abundant in rosacea skin, together with cathelicidin. The skin lesions (n = 5) of individuals with rosacea were biopsied and embedded into wax blocks. **(A)** Profound accumulation of inflammatory cells can be observed in the skin samples stained by HE. The pictures were taken under ×100, ×200, and ×400 magnification. **(B)** TLR2, KLK5, and cathelicidin are abundant in lesional skin of individuals with rosacea as detected by immunochemical staining; the pictures were taken under ×200 magnification. **(C)** Macrophages in lesional skin of individuals with rosacea as examined by immunohistochemistry with an antibody against CD68, and inflammatory reactions determined by staining with antibodies against TNF-α and IL-1β. The pictures were taken under ×200 magnification; ***P <0.001 (n = 5).

Microscopic examination of HE-stained sections clearly revealed large numbers of inflammatory cells infiltrating the dermis. The results of immunohistochemical staining showed higher expression of CD68, TLR2, KLK5, and cathelicidin in the lesions than that in the peri-lesion “normal” area (CD68 staining score of 2.13 *vs*. 24.16, P < 0.001; 0.91 *vs*. 5.94 for TLR2, P < 0.001; 2.948 *vs*. 15.83 for KLK5, P < 0.001, and 1.979 *vs*. 17.36 for cathelicidin, P < 0.001). Moreover, TNF-α and IL-1β expression was also higher in the lesion areas (1.80 *vs*. 14.17 for TNF-α, P < 0.001; 1.07 *vs*. 13.26 for IL-1β, P < 0.001) (**Figure 1**).

### Carvedilol Alleviates Skin Inflammation and Macrophage Infiltration in the Rosacea-Like Inflammation Induced by LL-37 in Mice

**Figure 2:**
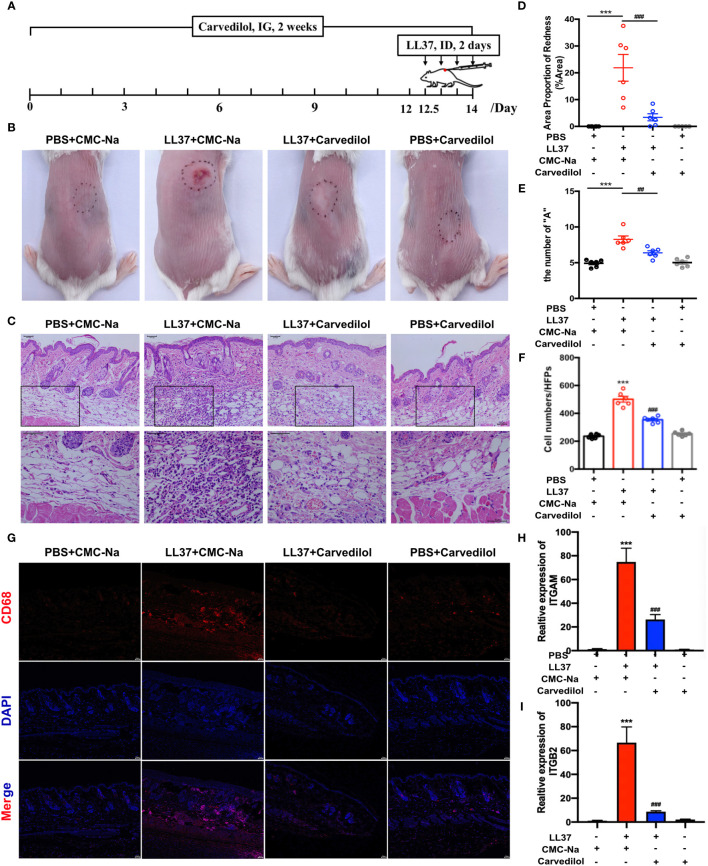
Carvedilol alleviates rosacea-like inflammation and macrophage infiltration induced by LL-37 in mouse skin. **(A)** PBS or cathelicidin LL-37 (50 μL, 320 mM) was injected intradermally into BALB/c mice to induce rosacea-like inflammation. Carvedilol (40 mg/kg) or CMC-Na was administrated by gavage for 14 days and animals observed 12 h after the last injection. **(B, C)** Skin manifestation of different groups. Obvious erythema was observed in the LL-37+CMC-Na group whereas pretreatment of carvedilol alleviated the reaction induced by LL-37 as shown in the LL-37+carvedilol group; conversely, carvedilol did not cause any obvious changes to the skin as shown in the PBS+carvedilol group. **(D)** The value of A* determined with a LAB colorimeter. **(E, F)** HE staining of skin samples from the four groups reveals obvious inflammation in the skin of the LL-37+CMC-Na group, which was significantly decreased in the LL-37+carvedilol group (n = 6). **(G)** Immunofluorescence staining of CD68 revealed that macrophages were accumulated in the LL-37+CMC-Na group, which was alleviated by pretreatment of carvedilol in the LL-37+carvedilol group. In comparison, PBS or carvedilol alone did not result in obvious changes (n = 4). The pictures were taken under ×100, ×200, and ×400 magnification. **(H, I)** Statistical analysis of the results of RT-qPCR of the genes for ITGAM, and ITGB2 (n = 4). ***P < 0.001, ^###^P < 0.001.

**Figure 3:**
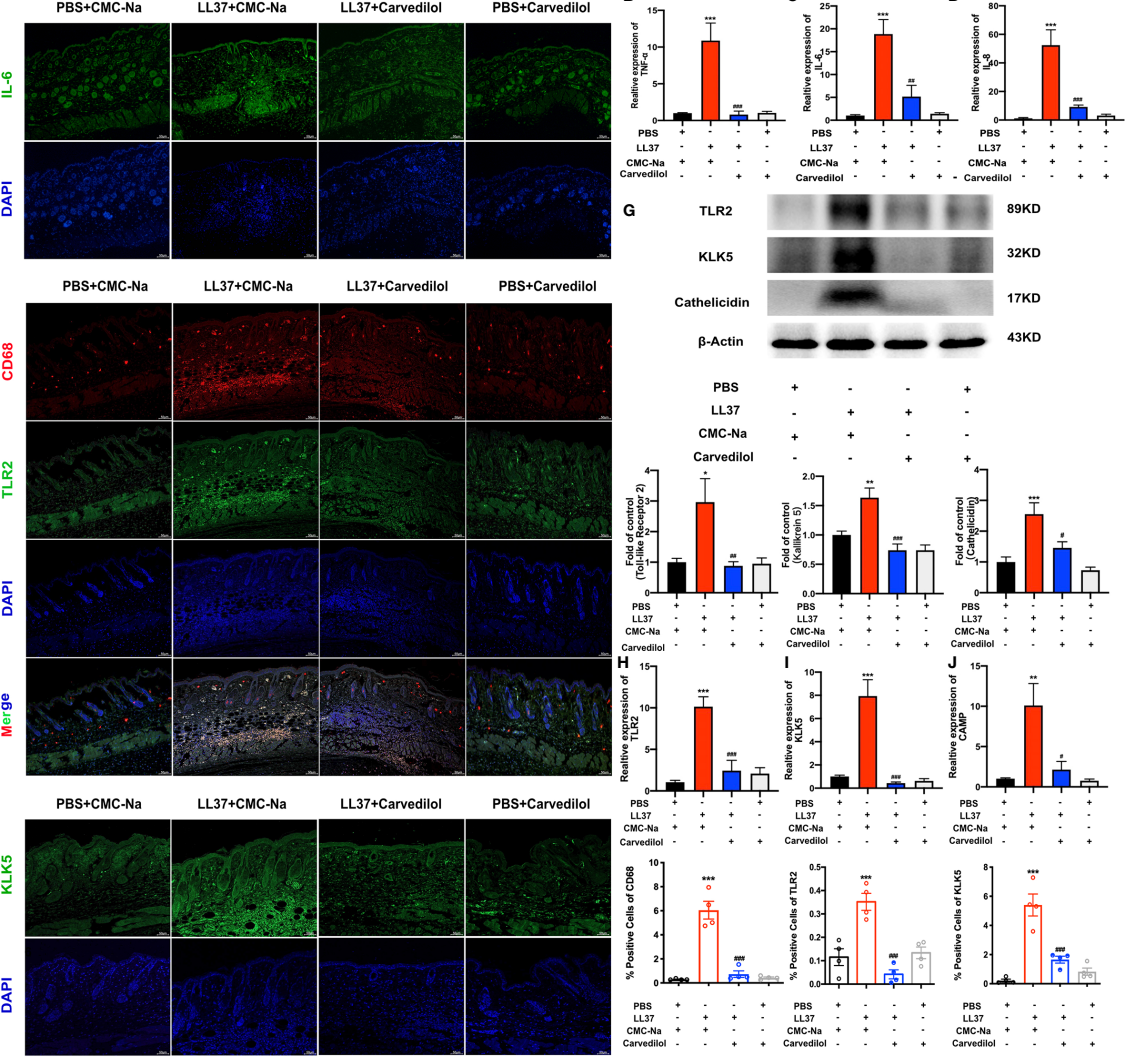
Carvedilol inhibits the expression of TLR2, KLK5, and cathelicidin and inflammatory factors in a mouse model of rosacea-like dermatitis. Immunofluorescence staining of IL-6 showed that inflammatory infiltration increase in ll-37+CMC-Na group, and decreased in carvedilol+LL-37 group (n=4). **(A)** Statistical analysis of the results of RT-qPCR of the genes for TNF-α, IL-6 and IL-8 (n=4). **(B–D)** Immunofluorescence staining of CD68 and TLR2 (n = 4). Carvedilol inhibits the expression of TLR2, KLK5, and cathelicidin in a mouse model of rosacea-like dermatitis. The expression of TLR2 and KLK5 was assessed by immunofluorescence, western blot, and RT-qPCR. The result shows that CD68 and TLR2 increased in the LL-37+CMC-Na group and decreased in the LL-37+carvedilol group, implying that the LL-37+CMC-Na group expressed more macrophages and TLR and that these could be down-regulated by carvedilol. Conversely, PBS or carvedilol alone did not cause obvious changes. **(E)** Immunofluorescence staining of KLK5 (n = 4). **(F)** The expression of KLK5 in the four groups was consistent with the results from **(E)**. Western blot analysis and quantitative results of TLR2, KLK5, and cathelicidin (n = 7). **(G)** Statistical analysis results of RT-qPCR of the genes for TLR2, KLK5, and cathelicidin (n = 4). **(H–J)** The expression of TLR2, KLK5, and cathelicidin protein and mRNA in the four groups also revealed similar trends as observed in **(E, F)**. *P < 0.05, **P < 0.01, ***P < 0.001, ^#^P < 0.05, ^##^P < 0.01, ^###^P < 0.001.

After four LL-37 injections, the mouse skin in the LL-37 group showed obvious erythema. Conversely, in the LL-37+carvedilol group, the color and range of erythema decreased, and the whole skin was nearly consistent with the surrounding skin (**Figures 2B, C**). Skin A* values as measured by colorimeter revealed a similar tendency (average values of PBS+CMC-Na, LL-37+CMC-Na, LL-37+carvedilol, and PBS+carvedilol group were 5.263, 8.900, 6.290, and 4.553, respectively) (**Figure 2D**). Histopathological results showed that inflammatory cell infiltration was significantly increased in the LL-37+CMC-Na group albeit reduced in the LL-37+carvedilol group, whereas no obvious changes were observed in the PBS+carvedilol group (average cell number per high-power field of PBS+CMC-Na, LL-37+CMC-Na, LL-37+carvedilol, and PBS+carvedilol groups was 236.4, 501.4, 354.4, and 254.4, respectively; P < 0.01) (**Figures 2E, F**). CD68 and IL-6 expression in mouse skin as determined by immunofluorescence indicated significantly increased macrophage aggregation and inflammatory infiltration in the LL-37+CMC-Na group but decreased aggregation in the LL-37+carvedilol group (**Figures 2G**, **3A**). The results of RT-PCR also revealed significantly up-regulated *ITGAM*, *ITGB2, TNFA*, *IL6* and *IL8*, gene expression in the LL-37+CMC-Na compared with the PBS+CMC-Na group (P < 0.05), albeit significantly down-regulated expression in the LL-37+carvedilol group (P < 0.05) (**Figures 2H–I** and **3B–D**).

### Carvedilol Inhibits TLR2 Expression in the Rosacea-Like Inflammation Induced by LL-37 in Mice

The results of CD68 and TLR2 immunofluorescence staining showed that in the LL-37+CMC-Na group, rosacea-like inflammatory skin expressed more TLR2 on the surface of aggregated macrophages whereas this was significantly reduced on the surface of macrophages in the LL-37+carvedilol group. A similar tendency was observed upon KLK5 immunofluorescence staining (**Figures 3E, F**). The results of western blotting and RT-qPCR of TLR2 and KLK5 further confirmed these results (**Figures 3G–J**) and demonstrated that cathelicidin was obviously secreted and expressed in the LL-37+CMC-Na group but was decreased in the LL-37+carvedilol group.

### Carvedilol Inhibits LPS-Induced Inflammation and TLR2 Expression in RAW 264.7 Cells

**Figure 4:**
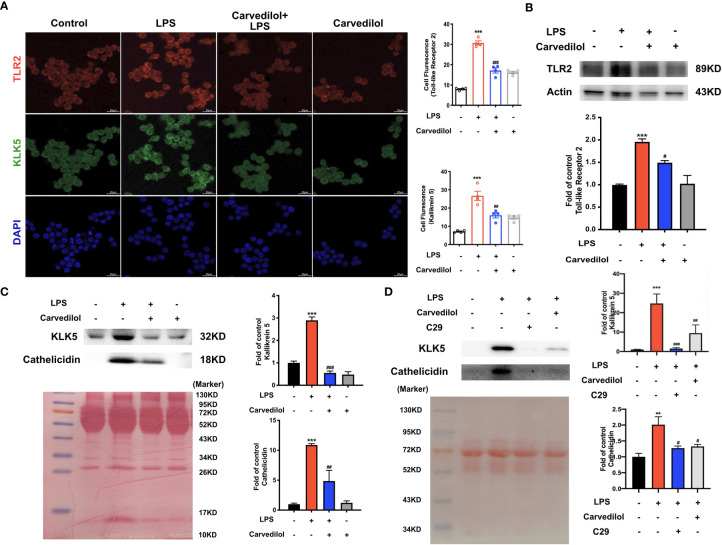
Carvedilol alleviates the expression of TLR2, KLK5, and cathelicidin in RAW 264.7 cells induced by LPS. **(A)** Immunofluorescence staining of TLR2 and KLK5. Following induction by LPS, the expression of TLR2 and KLK5 was significantly elevated, whereas expression was reduced by carvedilol pretreatment in the LPS+carvedilol group. **(B, C)** Western blot results of TLR2, KLK5, and cathelicidin expression showed an increase in the LPS group and decrease in the LPS+carvedilol group. **(D)** Western blot results of KLK5, and cathelicidin expression illustrated the same trend in the LPS+carvedilol and LPS+C29 group. **P < 0.01, ***P < 0.001, ^#^P < 0.05, ^##^P < 0.01, ^###^P < 0.001.

**Figure 5:**
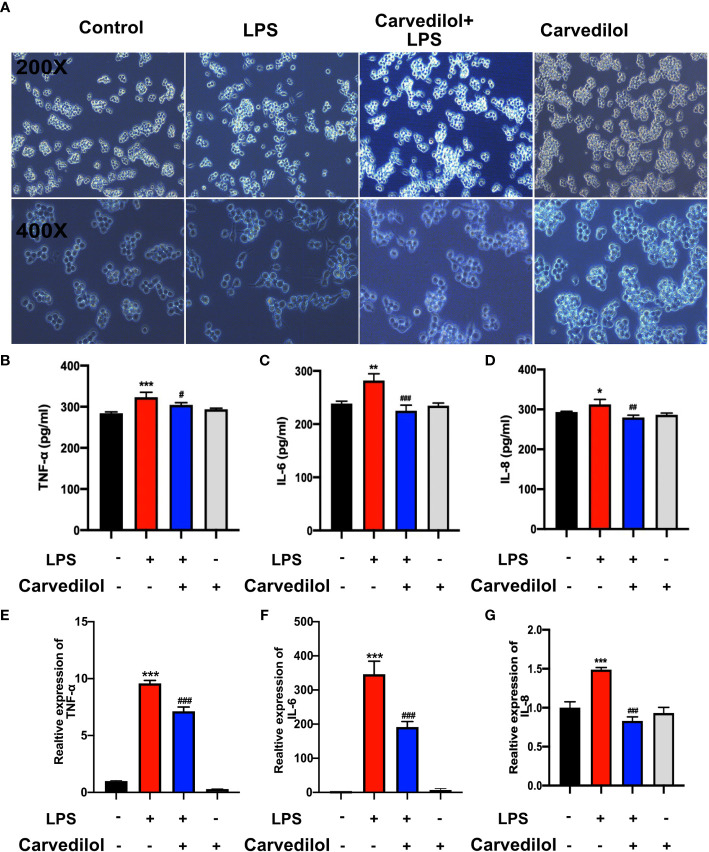
Carvedilol alleviates the inflammation in RAW 264.7 cells induced by LPS. The inflammatory model was established by 12 h LPS exposure with or without carvedilol pretreatment for 30 min. **(A)** Cell morphology and statistical results of the polarization ratio. In the LPS group, the shape of cells changed from round to spindle, indicating the occurrence of inflammation. With carvedilol pretreatment, the morphology of the cells did not change so much, mostly appearing as round shape. **(B–D)** Statistical analysis results of ELISA for inflammatory factor protein (including TNF-α, IL-6, and IL-8). **(E–G)** Statistical analysis results of RT-qPCR for inflammatory factor genes. The results further verified that LPS-induced inflammation could be abolished by carvedilol. *P < 0.05, **P < 0.01, ***P < 0.001, ^#^P < 0.05, ^##^P < 0.01, ^###^P < 0.001.

Following culture of RAW 264.7 cells with 1 μg/mL LPS for 12 h, TLR2, KLK5, and cathelicidin protein expression was significantly higher than that of the control group as shown by western blotting. Compared with the LPS group, TLR2, KLK5, and cathelicidin expression in carvedilol+LPS RAW 264.7 cells was relatively reduced. The immunofluorescence staining results were consistent with these findings (**Figures 4A–C**). Meanwhile, the expression of KLK5 and cathelicidin all decreased in carvedilol+LPS group, similar with results in C29+LPS group. (**Figure 4D**) The morphology of cells indicated that numerous cells were activated showing dendritic morphology, while fewer were observed in the carvedilol+LPS group. The results of ELISA and RT-qPCR further confirmed that gene expression of inflammatory factors, including TNF-α, IL-6, and IL-8 was enhanced in the LPS group compared to that in the carvedilol+LPS group (**Figure 5**).

### Oral Carvedilol Effectively Improves the Clinical Manifestation of Rosacea

**Figure 6:**
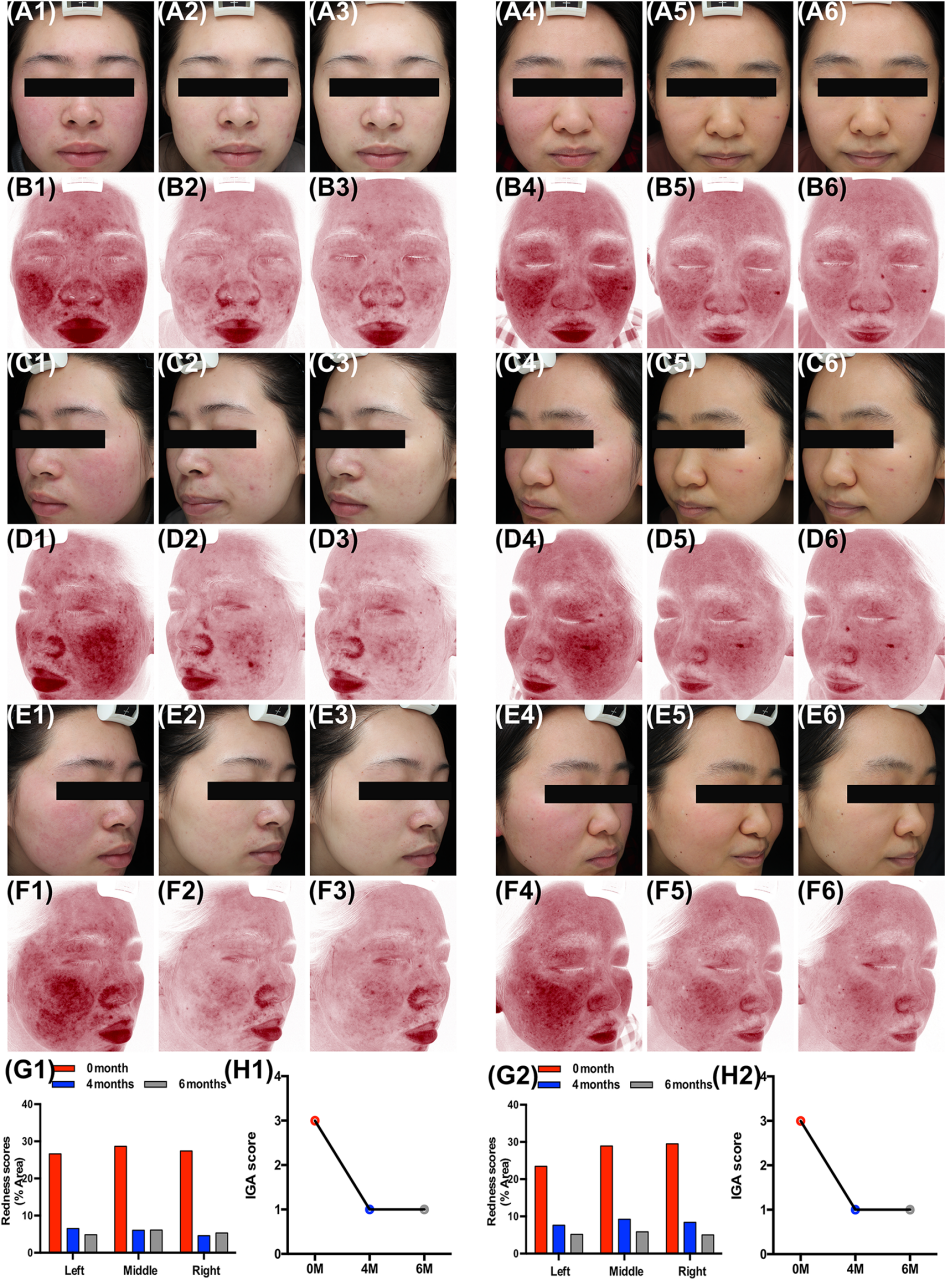
Oral carvedilol effectively alleviates rosacea. Patient 1: Clinical manifestation and red area images as analyzed by VISIA before the treatment (**A1**, **C1**, **E1**, and **B1**, **D1**, **F1**, respectively), at the four-month follow-up (**A2**, **C2**, **E2**, and **B2**, **D2**, **F2**, respectively), and at the six-month follow-up (**A3**, **C3**, **E3**, and **B3**, **D3**, **F3**, respectively). (**G1**) Before and six months after treatment. Image J was used to determine the percentage of redness in VISIA images. (**H1**) Investigator Global Assessment (IGA) score before and six months after treatment. Patient 2: Clinical manifestation and red area images as analyzed by VISIA before the treatment (**A4**, **C4**, **E4**, and **B4**, **D4**, **F4**, respectively), at the four-month follow-up (**A5**, **C5**, **E5**, and **B5**, **D5**, **F5**, respectively), and at the six-month follow-up (**A6**, **C6**, **E6**, and **B6**, **D6**, **F6**, respectively). (**G2**) Before and six months after treatment. Image J was used to determine the percentage of redness in VISIA images. (**H2**) IGA score before and six months after treatment.

Clinical presentation of two patients revealed that oral carvedilol significantly alleviated the clinical manifestation of rosacea after 4 and 6 months, showing a statistical difference in erythema index as determined using Image J. Prior to treatment, average redness scores of the left, middle, and right faces of two patients were 27.58 and 27.12, respectively. After four months of treatment, the average scores were reduced to 5.73 and 8.43, remaining at 5.35 and 5.36 after six months. In addition, the IGA score was consistent with previous results. (**Figure 6**) As carvedilol is routinely used in clinical practice for facial erythema treatment in rosacea, no RCT was included in this research. More clinical follow-up results are provided in **Supplement 1**.

## Discussion

In this study, we demonstrated that carvedilol reduced TLR2-induced inflammation in macrophages, which are involved in the pathogenesis of rosacea *in vivo* and *in vitro*. To our knowledge, this is the first study to elucidate the anti-inflammatory effects of carvedilol in rosacea treatment.

Systemic oral beta-adrenergic receptor blockers such as carvedilol are typically used to treat the flushing and erythema of rosacea, with carvedilol use first reported for refractory and persistent symptoms in 2011 (12) and subsequently recommended by several guidelines and consensuses especially for erythematotelangiectatic phenotypes (11, 17). Our clinical observations also confirmed these effects (18). Oral carvedilol (6.25 mg twice daily) obviously improved the clinical manifestation of rosacea during the 6-month follow-up, manifesting as less erythema in red area images as determined by VISIA and decreased A* value as detected by colorimetry and the IGA score.

The infiltration of innate cells including macrophages, mast cells, and neutrophils was associated with the immune reaction in rosacea (19–21). In 2015, neutrophils were reported in pustular regions of papulopustular rosacea (PPR) and phymatous rosacea (PhR) patients, while no evidence indicates it was involved in erythematotelangiectatic rosacea (ETR), by immunohistochemistry. Besides, macrophages in rosacea were supported by significant upregulation of cell markers of macrophages and upregulation of ITGB2 and ITGAM mRNA expression as well. In addition, it was also found in granulomatous and interfollicular areas in PPR, while with a more perivascular diffuse pattern in ETR (10). In 2011, it was reported that the increased density of mast cells was found by quantitative analysis of tryptase staining in all subtypes of rosacea, especially in PPR (21). Carvedilol was found to be effective for refractory ETR,with obvious alleviation of facial flushing and erythema. As a result, macrophages with a more perivascular diffuse pattern in ETR were regarded as one of the most important cells among the innate immune cells. While in our results, carvedilol was found to alleviate the infiltration of macrophages by Immunofluorescence and with the downregulation of expression of chemokines mRNAs, which further verify our hypothesis.

Keratinocytes react to pathogen-associated molecular patterns *via* TLRs (13, 14). Specifically, TLR2 and TLR4 have been shown to be overexpressed in rosacea skin (14, 22). In the present study we illustrated TLR2-mediated inflammation in rosacea by assessing the expression of TLR2, the serine protease KLK5, cathelicidin peptides, the macrophage-specific marker CD68, and related inflammatory factors using immunohistochemistry staining in the skin from patients with rosacea, in addition to immunofluorescence staining, western blot, and RT-qPCR on samples from rosacea-like mice, revealing that TLR2, KLK5, cathelicidin, and CD68 were all highly expressed in rosacea and rosacea-like lesions. In contrast, the TLR2/KLK5/cathelicidin pathway was down-regulated by pretreatment with carvedilol *in vitro*. Finally, the results in RAW 264.7 cells confirmed that the TLR2/KLK5/cathelicidin pathway could be enhanced in the rosacea-like inflammation mice model, whereas this was inhibited by pretreatment with carvedilol.

The rosacea-like dermatitis mouse model used in this study was generated as described previously (16, 23). Notably, 12 h following induction, rosacea-like erythema was enhanced as determined by observation with the naked eye, Image J analysis, and LAB colorimetry. The results of HE staining showed increased infiltration of inflammatory cells, especially macrophages, in the LL-37+CMC-Na group, which was inhibited by pretreatment with carvedilol. The results of RT-qPCR showed that carvedilol alleviated the elevated inflammatory cytokines (TNF-α, IL-6, and IL-8) and macrophage chemotactic factors (ITGAM, ITGB2) of rosacea-like skin, indicating that inflammation and macrophages are inhibited. Our results therefore illustrated that at the animal level, carvedilol could relieve erythema and inflammation of rosacea-like skin. Notably, the results in animals were markedly similar to those in patients. Together, these findings indicate that rosacea can induce inflammation progression and macrophage chemotaxis whereas these are reduced by carvedilol treatment.

Moreover, TLR2, KLK5, cathelicidin peptides, and CD68 were highly expressed in both patients with rosacea and rosacea-like animals. At the animal level, we verified that carvedilol could down-regulate the TLR2/KLK5/cathelicidin pathway. Double-label immunofluorescence staining revealed that carvedilol could decrease levels of TLR2, which was mainly expressed on macrophages, in the skin of rosacea-like mice. Furthermore, we demonstrated that LPS-induced activation of the TLR2/KLK5/cathelicidin pathway in RAW 264.7 cells could be down-regulated by carvedilol.

This study had several limitations. In particular, no pathological evidence was available with regard to an association between the TLR2/KLK5/cathelicidin changes in macrophages and clinical improvement in patients with rosacea because it is difficult to persuade the patients to undergo biopsy of facial skin owing to the risk of scar formation.

In conclusion, we present the first report that carvedilol can alleviate the inflammatory reaction in rosacea *via* the TLR2/KLK5/cathelicidin pathway in macrophages. Additional studies are needed to further clarify the intrinsic mechanism and the relationship between inflammatory status, adrenergic receptors, TLRs, and inflammatory status in rosacea.

## Data Availability Statement

The original contributions presented in the study are included in the article/**Supplementary Material**. Further inquiries can be directed to the corresponding authors.

## Ethics Statement

The studies involving human participants were reviewed and approved by the First Affiliated Hospital of Nanjing Medical University. The patients/participants provided their written informed consent to participate in this study. The animal study was reviewed and approved by Nanjing Medical University Animal Care and Use Committee. Written informed consent was obtained from the individual(s) for the publication of any potentially identifiable images or data included in this article.

## Author Contributions

JZ completed the majority of experiment and wrote the draft. PJ and LS participated in the animal studies. YYL, YXL, ML, and MT prepared the biological samples and contributed to analyzing the data. XW collected the information of patients. LH supervised the whole productions. YX and WL designed and supervised the study and wrote the final version of the manuscript. All authors contributed to the article and approved the submitted version.

## Funding

This research was supported by the Chinese National Natural Science Foundation (81301384), the Priority Academic Program Development of Jiangsu Higher Education Institutions (No. JX10231801), and the Natural Science Foundation of Jiangsu Province [BK20181363].

## Conflict of Interest

The authors declare that the research was conducted in the absence of any commercial or financial relationships that could be construed as a potential conflict of interest.

## Abbreviations

HE, hematoxylin–eosin; i.d., intradermal; i.g., intragastric; IGA, Investigator Global Assessment; KLK5, kallikrein-related peptidase 5; LPS, lipopolysaccharide; NRS, National Rosacea Society; TLR2, Toll-like receptor 2.
