# Corrigendum: A Novel Mechanism of Carvedilol Efficacy for Rosacea Treatment: Toll-Like Receptor 2 Inhibition in Macrophages

**DOI:** 10.3389/fimmu.2022.881277

**Published:** 2022-03-14

**Authors:** Jiawen Zhang, Peiyu Jiang, Lei Sheng, Yunyi Liu, Yixuan Liu, Min Li, Meng Tao, Liang Hu, Xiaoyan Wang, Yanjing Yang, Yang Xu, Wentao Liu

**Affiliations:** ^1^ Department of Dermatology, The First Affiliated Hospital of Nanjing Medical University, Nanjing, China; ^2^ Jiangsu Key Laboratory of Neurodegeneration, Department of Pharmacology, Nanjing Medical University, Nanjing, China; ^3^ Department of Dermatology, The Second Affiliated Hospital of Nanchang University, Nanchang, China; ^4^ Jiangsu Key Laboratory of Oral Disease, Nanjing Medical University, Nanjing, China

**Keywords:** rosacea, carvedilol, macrophage, TLR2, KLK5, cathelicidin

In the original article, there was a mistake in **Figure 1** as published. In **Figure 1A**, the y-axis of the top graph was labelled “CD68 staining score”, when it should have been labelled “TLR2 staining score”. The corrected **Figure 1** appears below.

**Figure 1:**
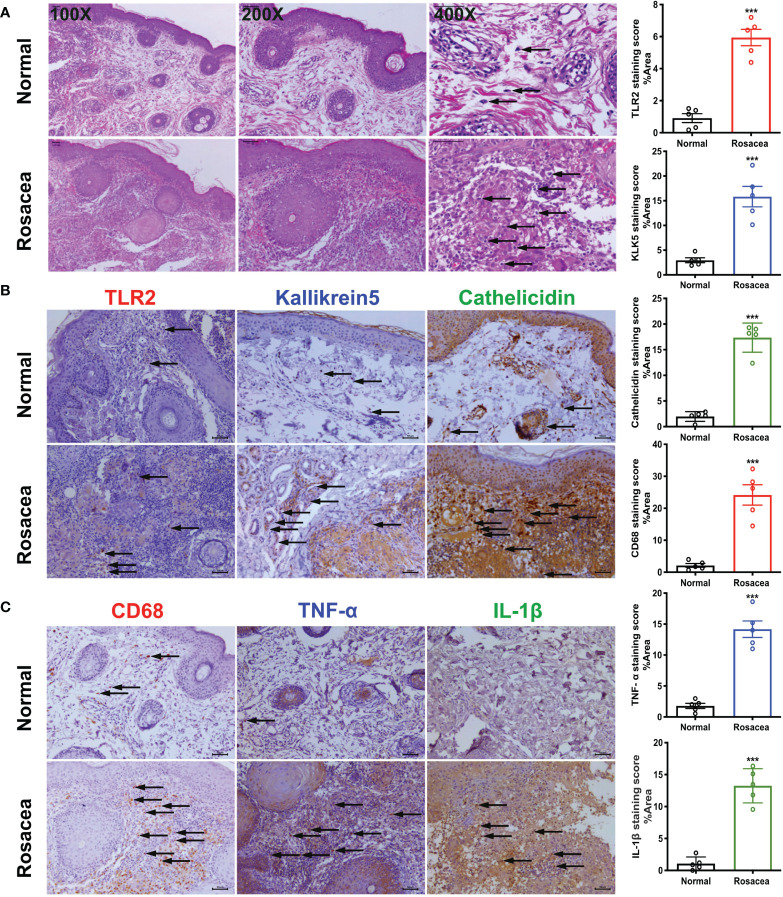
Inflammatory cells especially macrophages are abundant in rosacea skin, together with cathelicidin. The skin lesions (n = 5) of individuals with rosacea were biopsied and embedded into wax blocks. **(A)** Profound accumulation of inflammatory cells can be observed in the skin samples stained by HE. The pictures were taken under ×100, ×200, and ×400 magnification. **(B)** TLR2, KLK5, and cathelicidin are abundant in lesional skin of individuals with rosacea as detected by immunochemical staining; the pictures were taken under ×200 magnification. **(C)** Macrophages in lesional skin of individuals with rosacea as examined by immunohistochemistry with an antibody against CD68, and inflammatory reactions determined by staining with antibodies against TNF-α and IL-1 β. The pictures were taken under ×200 magnification; ***P <0.001 (n = 5).

The authors apologize for this error and state that this does not change the scientific conclusions of the article in any way. The original article has been updated.

## Publisher’s Note

All claims expressed in this article are solely those of the authors and do not necessarily represent those of their affiliated organizations, or those of the publisher, the editors and the reviewers. Any product that may be evaluated in this article, or claim that may be made by its manufacturer, is not guaranteed or endorsed by the publisher.

