# Supplementary material for: A Novel Mechanism of Carvedilol Efficacy for Rosacea Treatment: Toll-Like Receptor 2 Inhibition in Macrophages
Source: Front Immunol. 2021 Jul 12;12:609615. doi: 10.3389/fimmu.2021.609615 (PMC8311793; doi:10.3389/fimmu.2021.609615)
Supplement: Supplementary file 1 [file Image_1.pdf]

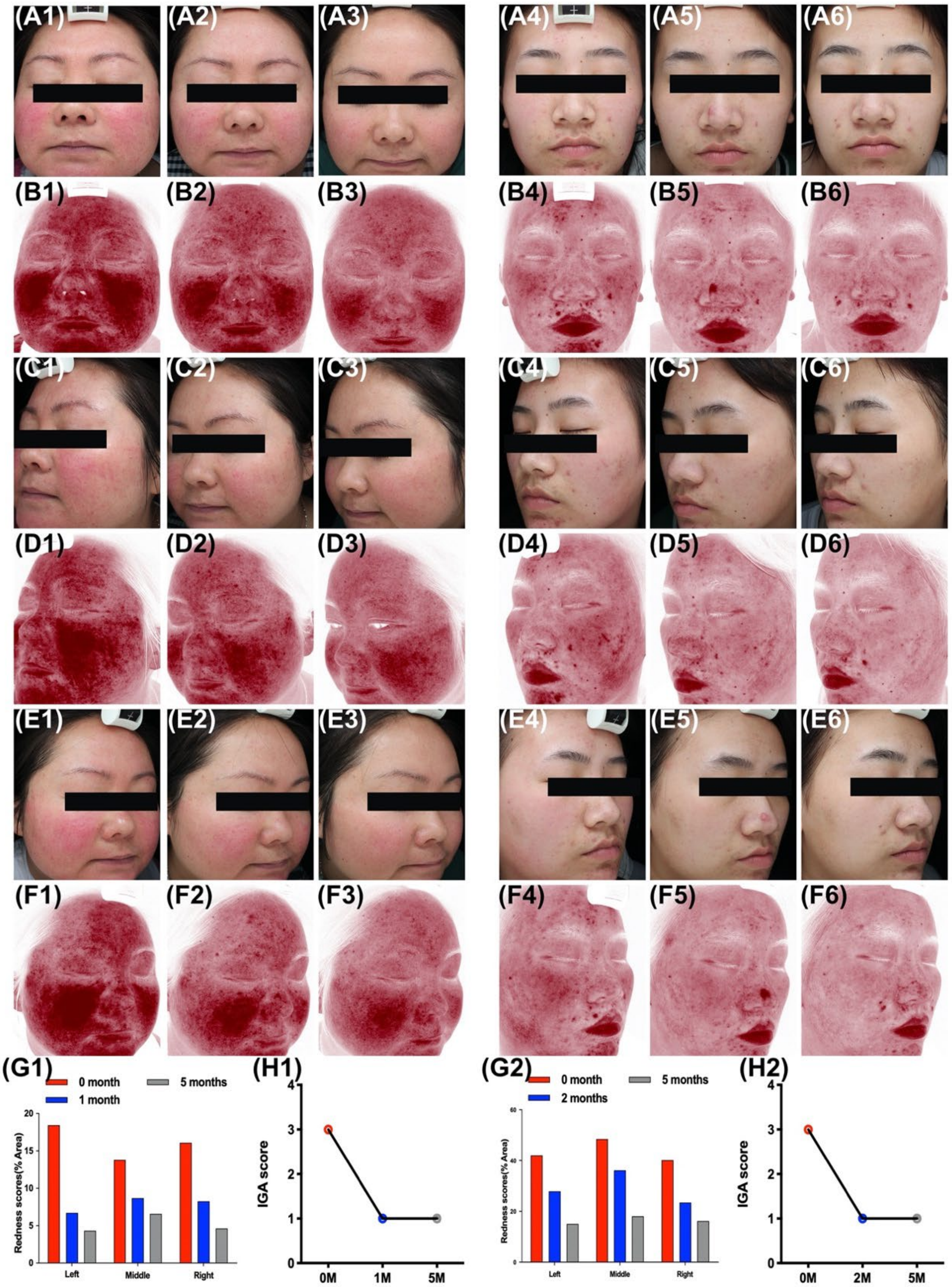

Supplementary Fig. S1. Oral carvedilol effectively alleviates rosacea. Patient 3: Clinical manifestation and red area images as analyzed by VISIA before the treatment (A1, C1, E1, and B1, D1, F1, respectively), at the one-month follow-up (A2, C2, E2, and B2, D2, F2, respectively), and at the five-month follow-up (A3, C3, E3, and B3, D3, F3, respectively). (G1) Before and five months after treatment. Image J was used to determine the percentage of redness in VISIA images. (H1) Investigator Global Assessment (IGA) score before and five months after treatment. Patient 4: Clinical

manifestation and red area images as analyzed by VISIA before the treatment (A4, C4, E4, and B4, D4, F4, respectively), at the two-month follow-up (A5, C5, E5, and B5, D5, F5, respectively), and at the five-month follow-up (A6, C6, E6, and B6, D6, F6, respectively). (G2) Before and five months after treatment. Image J was used to determine the percentage of redness in VISIA images. (H2) IgA score before and five months after treatment.
