# Supplementary material for: A Novel Mechanism of Carvedilol Efficacy for Rosacea Treatment: Toll-Like Receptor 2 Inhibition in Macrophages
Source: Front Immunol. 2021 Jul 12;12:609615. doi: 10.3389/fimmu.2021.609615 (PMC8311793; doi:10.3389/fimmu.2021.609615)
Supplement: Supplementary file 2 [file Image_2.pdf]

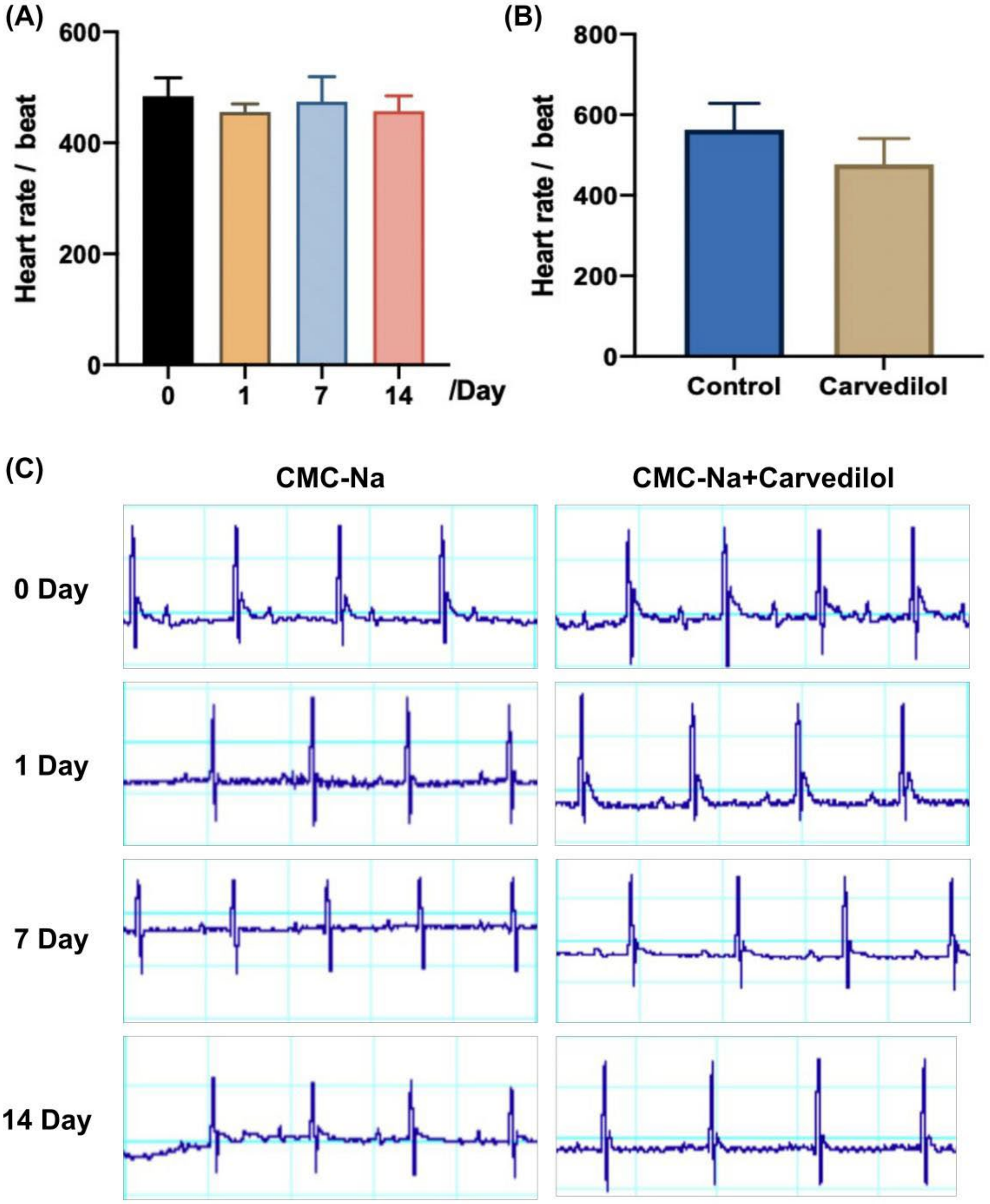

Supplementary Fig. S2. No obvious changes were observed in heart rate and electrocardiogram in mice with carvedilol (40 mg/kg) gavage. (A) The heart rate of mice before and 1,7,14 days after carvedilol gavage. (B) The heart rate in CMC-Na group and CMC-Na+Carvedilol group 14 days after gavage. (C)The electrocardiogram of mice in CMC-Na group and CMC-Na+Carvedilol group.
